# Role of histone-lysine N-methyltransferase 2D (KMT2D) in MEK-ERK signaling-mediated epigenetic regulation: a phosphoproteomics perspective

**DOI:** 10.3389/fbinf.2025.1683469

**Published:** 2025-11-18

**Authors:** Sreeshma Ravindran Kammarambath, Leona Dcunha, Athira Perunelly Gopalakrishnan, Amal Fahma, Neelam Krishna, Altaf Mahin, Samseera Ummar, Prathik Basthikoppa Shivamurthy, Inamul Hasan Madar, Rajesh Raju

**Affiliations:** Centre for Integrative Omics Data Science, Yenepoya (Deemed to be University), Mangalore, Karnataka, India

**Keywords:** KMT2D, MEK-ERK signaling, phosphoproteomics, kabuki syndrome, epigenetic regulation, phosphorylation sites

## Abstract

**Introduction:**

Histone-lysine N-methyltransferase 2D (KMT2D) is an H3K4 methyltransferase and a potential tumor suppressor with a crucial role in regulating gene expression. Its dysregulation has been implicated in developmental disorders and several types of cancers. Despite this, the molecular mechanisms that govern its activity remain largely elusive. Among these, post-translational modifications, especially phosphorylation, serve as an essential regulator, fine-tuning KMT2D stability, localization and functional interactions for maintaining cellular homeostasis. With over 173 phosphorylation sites reported, KMT2D is significantly regulated by kinases and exploring its phospho-regulatory network based on targeted *in vitro* approaches is challenging.

**Methods:**

We systematically curated and integrated the global phosphoproteomic datasets, along with their corresponding experimental conditions, to comprehensively identify the phosphorylation events reported for KMT2D. The site exhibiting the highest frequency of detection across these datasets is considered the predominant phosphorylation site. To investigate its functional significance, we analyzed the proteins and their phosphorylation sites that are differentially co-regulated with the predominant site, as well as its associated upstream kinases and interacting proteins.

**Results:**

Among the 173 reported phosphorylation sites representing KMT2D, Serine 2274 (S2274) emerged as the predominant site being detected in over 42% of diverse mass spectrometry-based phosphoproteomics datasets. This site lies within one of KMT2D’s unique “*LSPPP*” motifs, suggesting a potential regulatory role. Detailed investigation on the differentially co-regulated protein phosphosites revealed the phosphorylation of KMT2D at S2274 is consistently and positively co-regulated with MAPK1/ERK2 activation, as well as with the proteins involved in the MAPK cascade, epigenetic regulation and cell differentiation. Notably, ERK2 was predicted as an upstream kinase targeting S2274, suggesting that KMT2D S2274 functions as a potential downstream effector of MEK-ERK signaling pathway, potentially linking to epigenetic regulation and cell differentiation. Further, our results highlighted a potential mechanistic link between disrupted phosphorylation at S2274 and the pathogenesis of Kabuki syndrome.

**Discussion:**

This study delineates the phosphoregulatory network of KMT2D, positioning it as a dynamic epigenetic effector modulated by MEK-ERK signaling, with broader implications for cancer and developmental disorders.

## 1 Introduction

In 1997, Prasad et al. identified a novel gene using the ALL1 SET domain as a probe, initially naming it the ALL1-related gene (ALR), which was later designated as Histone-lysine N-methyltransferase 2D (KMT2D) (Prasad et al., 1997). It is also known as MLL4 and MLL2 and belongs to the family of mammalian histone H3 lysine 4 (H3K4) methyltransferases (Froimchuk et al., 2017). KMT2D is one among the six Set1-like (COMPASS) H3K4 methyltransferases present in mammals, along with KMT2A (or MLL1), KMT2B (or MLL2), KMT2C (or MLL3), KMT2D (or MLL4, ALR, and sometimes MLL2), KMT2F (or SET1A), and KMT2G (or SET1B) (Mohan et al., 2011). The KMT2D gene encodes a 5537 amino acid protein, which has a molecular weight of 593,389 Da. The human ortholog of KMT2D is situated on chromosome 12q13.12 (Froimchuk et al., 2017). KMT2D contains a catalytically active SET domain, five PHD fingers, potential zinc fingers and a long stretch of glutamines interrupted by hydrophobic residues (Prasad et al., 1997). The KMT2 proteins reside in large, multi-subunit complexes composed of unique interacting proteins. The KMT2D protein contains two clusters of plant homeodomain (PHD) motifs located in the N-terminal region, each containing three PHDs and a catalytically active SET domain at the C-terminus (Ruthenburg et al., 2007). The PHDs in the second cluster (PHD4-6) can bind to H4 tails on nucleosomes in vitro, which may be essential for cell-driven nucleosome methylation (Dhar et al., 2012). Adjacent to the SET domain are PHD and FY-rich N/C-terminal (FYRN and FYRC) domains. Additionally, the protein also contains a high mobility group (HMG-I) and nine nuclear receptor interaction motifs (LXXLLs) (Rao and Dou, 2015). Structural analysis has further revealed that conserved tyrosine residues within the SET domain specifically Y5426 and Y5512 in human KMT2D are indispensable for its histone H3K4 methyltransferase activity *in vitro* (Jang et al., 2017). The PHD4-6 domains of KMT2D bind to both unmethylated and asymmetrically di-methylated arginine 3 on histone H4 (H4R3me0 and H4R3me2a), involved together in protein arginine methyltransferase activity (Dhar et al., 2012).

KMT2D plays an important role in transcription regulation and cellular metabolism. It plays a key role in driving transcriptome changes during adipogenesis and the transdifferentiation of pre-adipocytes into myocytes. Consequently, earlier studies have also provided evidence of the importance of muscle adipose tissue development (Lee et al., 2013). It is also essential for the expression of cell-type-specific genes during neuronal and osteoblast differentiation (Dhar et al., 2012; Mu et al., 2017). The functional impact of KMT2D has been diverse from hepatic circadian rhythm to tumour suppressor. KMT2D is an epigenetic regulator of the hepatic circadian clock which acts as a transcriptional coactivator of the circadian TFs retinoid-related orphan receptor (ROR)-α and -γ (Kim et al., 2015). Additionally, studies have identified KMT2D as a tumour suppressor, specifically in follicular lymphoma and diffuse large B-cell lymphoma (Zhang et al., 2015; Ortega-Molina et al., 2015). KMT2D, along with NCOA6 acts as a coactivator of tumor suppressors and is required for the expression of endogenous p53 target genes in response to the DNA-damaging agent to the cancer cells such as Doxorubicin (Lee et al., 2009). Beyond its role as tumor suppressor, Ng et al. (2010) reported that the exome sequencing of patients with Kabuki syndrome revealed loss of function mutations in the KMT2D gene, linking these mutations to the development of the syndrome (Ng et al., 2010). Approximately 80% of mutations in individuals with Kabuki syndrome have predominant mutations specifically in the KMT2D gene (Khodaeian, 2021). Kabuki syndrome is a disorder marked by unique facial features, heart and skeletal abnormalities, immune system defects, and mild to moderate intellectual disability (Niikawa et al., 1981). Apart from Kabuki syndrome mutation, KMT2D has been found to be associated with congenital heart disease (Zaidi et al., 2013). KMT2D mutations are frequently observed across multiple cancers, which include malignancy of brain, lymph nodes, blood, lungs, large intestine and endometrium (Rao and Dou, 2015), bladder, lung and endometrial cancers (Kandoth et al., 2013), prostate cancer (CRPC) (Grasso et al., 2012), lung adenocarcinomas and squamous cell carcinomas (Campbell et al., 2016), acute lymphoblastic leukemia (Mar et al., 2012), oesophageal carcinoma (Dhar and Lee, 2021).

Protein phosphorylation is a significant post-translational modification (PTMs) that plays an important role in regulating the diverse cellular and molecular functions of proteins (Kim et al., 2014). Advances in mass spectrometry with diverse separation techniques enable simultaneous analysis of expression levels for over thousands of proteins (Zhu et al., 2003). Mass Spectrometry-based Proteomic analysis has paved a revolutionary role in the identification of peptides, proteins, and their PTMs, especially phosphorylated peptides, and their characterization, quantification, and applications in clinical theragnostic (Hyeon et al., 2025; Madar et al., 2017). Through mass spectrometry-based phosphoproteomic data analysis, this study reveals a phosphoregulatory network that positions KMT2D as a dynamically phosphorylated epigenetic effector modulated by MEK–ERK signaling, with broad implications in cancer and developmental disorders.

## 2 Methodology

### 2.1 Assembling the global phosphoproteomics datasets of KMT2D

An extensive and systematic literature search on PubMed was conducted using the keywords “phosphoproteomics” or “phosphoproteome”, excluding “Plant” and “Review” articles, to map the human cellular global phosphoproteome datasets containing phosphorylation sites of KMT2D. We screened the literature to assemble human cell line-based global high-throughput phosphoproteome datasets with phosphorylation sites. These datasets were collected from studies employing diverse experimental platforms. Details on the quantification approach (label-free, TMT, SILAC), mass spectrometry instruments (e.g., Orbitrap), enrichment methods (TiO2, Fe-IMAC), and Class I site identification criteria are provided in Supplementary Material 1. These parameters represent the main sources of technical variability in phosphoproteomic workflows and were considered during dataset curation to ensure a consistent level of analytical comparability across studies.

We only considered the phosphorylation sites defined by a localization probability of ≥75% or an A-score of ≥13. Phosphorylation sites meeting either of these criteria were classified as class I sites, reflecting high confidence in the accurate assignment of phosphorylation to specific residues within the peptides. All these phosphoproteome datasets were derived from LysC and/or Trypsin-digested peptide analysis.

The datasets were subsequently classified into two: (i) quantitative differential datasets, which compare test biological or experimental conditions against corresponding controls, and (ii) qualitative profile datasets, where test and control conditions are treated as independent datasets. These datasets were categorized based on the individual phosphorylation site enrichment methods used in each study (STY/ST/Y phosphorylation sites) (Priyanka et al., 2024). Further, the class 1 phosphorylation sites are classified into upregulated and downregulated based on their fold change and p-value (<0.05). The phosphorylation sites whose fold change value is ≥1.3 are selected as upregulated, and ≤0.76 are selected as downregulated.

**FIGURE 1:**
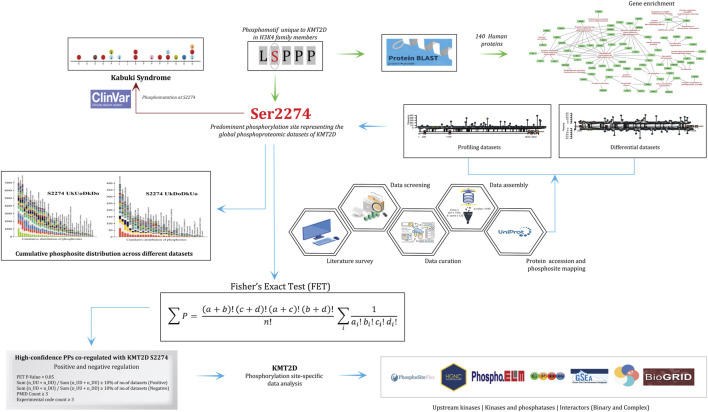
Flowchart detailing the methodological framework for analyzing the phosphorylation site S2274 in KMT2D. It includes data screening, literature survey, data curation, and assembly using various bioinformatics tools. Key processes depicted are protein profiling, differential datasets, and cumulative phosphosite distribution of phosphorylation sites. The analysis connects to clinical data with references to Kabuki Syndrome. Statistical evaluation using Fisher's Exact Test (FET) was performed to identify the differentially co-regulated high-confidence protein phosphosites with S2274, which were subsequently examined for their representation within the upstream kinases and predicted interactors, enabling functional interpretation of KMT2D signaling dynamics.

Each protein was mapped to its corresponding gene symbol based on the HGNC (downloaded on 30.05.2023) and to its corresponding UniProt (13.04.2023) (UniProt, 2023) accessions using our in-built mapping tool to ensure consistent and standardized annotation. We conducted the analysis using the methodologies outlined in (Sanjeev et al., 2024). The overall workflow used in this study is outlined in Figure 1.

### 2.2 Identification of the predominant phosphorylation sites representing KMT2D

To identify the predominantly represented phosphorylation sites from the human cellular qualitative phosphoproteomic datasets enriched for S/T/Y residues, the phosphorylation sites were ranked according to their frequency of detection across the compiled datasets. This approach enabled prioritization of the consistently observed phosphorylation events. Among them, the phosphorylation sites with the highest frequency were selected as the predominant phosphorylation sites in global phosphoproteomics. A lollipop plot was generated using the R/Bioconductor package trackViewer (Ou and Zhu, 2019) to visualize the phosphorylation sites representing KMT2D.

#### 2.2.1 Investigating the possible co-occurrence of phosphorylation sites representing KMT2D

To investigate the mutual relationships among phosphorylation sites within KMT2D, a co-occurrence analysis was performed. In particular, we looked into the co-differential regulatory pattern of phosphorylation sites within KMT2D to determine the co-regulated patterns of the identified KMT2D phosphorylation sites. We independently determined the U(K1)U(K2), U(K1)D (K2), D (K1)D (K2), and D (K1)U(K2) frequencies corresponding to each KMT2D phosphorylation site in each differential data set where multiple phosphorylation sites were found. The (K1) and (K2) represent the two different phosphorylation sites of KMT2D. To access the co-regulation patterns, we applied the ratio ∑(nU(K1)U(K2) + nD (K1)D (K2))/∑(nU(K1)D (K2) + nD (K1)U(K2)) to evaluate their positive co-regulation and ∑(nU(K1)D (K2) + nD (K1)U(K2))/∑(nU(K1)U(K2) + nD (K1)D (K2)) to evaluate their negative co-regulation. A positive co-regulation frequency >3 is considered significant for positive co-occurrence between KMT2D phosphorylation sites, and a negative co-regulation frequency <3 is considered significant for negative co-occurrence between KMT2D phosphorylation sites, given the low abundance of differential datasets where multiple phosphorylation sites are detected together. The degree of dependency between each phosphorylation site is determined through this co-occurrence plot to observe how closely they are interlinked. The phosphorylation sites that are upregulated and downregulated together can be understood from the plot.

### 2.3 Analysis of the protein phosphosites in other proteins (PPs) that differentially co-regulated with predominant phosphorylation sites of KMT2D

To analyze the phosphorylation site in other proteins (PPs) that are positively and negatively co-regulated with KMT2D, we grouped them based on their co-regulation patterns in various differential quantitative datasets. To illustrate the expression patterns of predominant phosphorylation sites across different studies, we first examined the differential expression between KMT2D phosphorylation site pairs. We paired KMT2D phosphorylation sites with other protein phosphosites to visualize the differential co-regulation patterns among them. Each category label (e.g., UkUo, DkDo, UkDo, DkUo) denotes the combined regulation status of two factors. The first component represents the expression level of KMT2D, where “Uk” indicates upregulation and “Dk” indicates downregulation. The second component corresponds to the expression status of PPs, protein phosphosites are the phosphosites in other proteins which are differentially co-regulated with the KMT2D sites, with “Uo” signifying their upregulation and “Do” signifying their downregulation. The positively co-differentially regulated PPs and negatively differentially regulated PPs with KMT2D are labelled as UkUoDkDo (positively co-regulated) and UkDoDkUo (negatively co-regulated), respectively.

A Fisher’s Exact Test (FET) was employed by constructing a contingency table for the corresponding KMT2D and other protein sites.

Fisher’s exact test (FET):

$$\sum P=\frac{\left(a+b\right)!\left(c+d\right)!\left(a+c\right)!\left(b+d\right)!}{n!}\;\sum_{i}\frac{1}{a_{i}!b_{i}!c_{i}!d_{i}!}$$

In this equation, ‘**a** (n_0k0o)’ denotes the number of experimental conditions in which neither of the sites were detected; ‘**b** (n_Uk0+n_Dk0+n_0Uo + n_0Do)’ denotes the number of experimental conditions where only one of the two sites were detected (either up/downregulated), while the other was not detected; ‘**c** (n_UkDo + n_DkUo)’ denotes the number of experimental conditions showing negative co-regulation between the two sites; ‘**d** (n_UkUo + n_DkDo)’ denotes the number of experimental conditions showing positive co-regulation between the two sites. Based on these values, a contingency table was constructed, and Fisher’s Exact Test (FET) was applied to compute the statistical significance (P value) of co-regulation patterns.

To reduce the dataset biases, high-confidence phosphorylation site pairs were identified from the FET-derived contingency table of proteins on the following criteria: (i) one-side FET score (p-value) < 0.05, (ii) ratio meeting at least 10% of total differential datasets, and (iii) detection in at least three independent studies and three unique experimental conditions (Supplementary Material 4).

This pair of protein phosphosites satisfying these thresholds was used for the downstream analysis to explore their potential role in distinct biological processes, signaling pathways, protein-protein interactions, and kinase-substrate relationships of phosphorylation site (Mahin et al., 2025).

### 2.4 Deriving the upstream kinases and interactor proteins of KMT2D

For deriving the upstream kinases, phosphatases, co-differentially regulated proteins, and interactors (binary and complex), the following databases and prediction tools are used. The experimentally known protein-protein interactors were extracted from BIND (Bader et al., 2003), BioGRID (Oughtred et al., 2021), HPRD (Keshava Prasad et al., 2009), CORUM (downloaded on 03.03.2023) (Tsitsiridis et al., 2023), ConsensusPathDb release 35 (downloaded on 22.05.2023) (Kamburov and Herwig, 2022), and RegPhos 2.0 (downloaded on 24.05.2023) (Huang et al., 2014). The experimentally validated and predicted upstream kinases for KMT2D were identified using multiple kinase prediction tools, including NetworKIN (accessed on 04.01.2023) (Linding et al., 2008) and AKID (accessed on 24.05.2023) (Parca et al., 2019), as well as those obtained from a high-throughput *in vitro* screen of 385 kinases available in iKiP-DB (Mari et al., 2022), phosphositePlus (downloaded on 22 May 2023) (Hornbeck et al., 2015), and kinases/substrates derived from the study by Johnson et al. (2023) with a cutoff of 90% and above (Johnson et al., 2023).

### 2.5 Functional enrichment analysis and data visualization

The functional gene enrichment analysis of the PPs that are either positively and negatively co-regulated with KMT2D was analyzed by using g:Profiler tool (Raudvere et al., 2019) and DAVID bioinformatics (Sherman et al., 2022). Multiple sequence alignment (MSA) was performed using Clustal Omega (Sievers and Higgins, 2018). Cytoscape (Shannon et al., 2003), PathVisio 3 (Kutmon et al., 2015), RAWGraphs 2.0 (Rawgraph, 2013), Adobe Illustrator (2020), and BioRender (2023) (Biorender, 2025) were used for the visualization of the results and pathways.

## 3 Result and discussion

### 3.1 Comparative conservation analysis of H3K4 methyltransferase family members

Histone modifications are key regulators of chromatin accessibility and gene expression, which are fundamentally associated with human development and disease. Among these, the H3K4 methyltransferase family, including, KMT2A (MLL1), KMT2B (MLL2), KMT2C (MLL3), KMT2D (MLL4/ALR, sometimes referred to as MLL2), KMT2E (inactive), KMT2F (SET1A) and KMT2G (SET1B) are highly conserved and ubiquitously expressed across human tissues. Recent studies show that KMT2D is associated with gene expression regulation and many signaling pathways through multiple mechanisms, unraveling its crucial role in regulating cell proliferation and maintaining cell cycle homeostasis (Mohan et al., 2011; Wang et al., 2025). Although members of the H3K4 methyltransferase family differ in their domain architecture, they all share the conserved catalytic SET domain responsible for mediating H3K4 methylation. Individual family members display distinct substrate preferences, catalyzing mono-, di-, or tri-methylation at enhancers and promoters. Among them, KMT2D functions primarily as an H3K4 mono- and di-methyltransferase, depositing active enhancer marks that drive lineage-specific transcriptional programs (Jang et al., 2017). Together, these observations illustrate the strong evolutionary conservation of catalytic residues within the KMT2 family, reinforcing their shared mechanistic role in chromatin regulation while also emphasizing the unique enhancer-associated functions of KMT2D in cell proliferation and cell-cycle homeostasis.

Comparative studies show that KMT2D in particular is associated with gene expression regulation and many signaling pathways through multiple mechanisms, unraveling its crucial role in regulating cell proliferation and maintaining cell cycle homeostasis. To further explore evolutionary conservation within the H3K4 methyltransferase family, a multiple sequence alignment was conducted using Clustal Omega. Notably, multiple “LSPPP” phosphomotif were found uniquely enriched exclusively in KMT2D and not detected in any other H3K4 methyltransferase (KMTs) family, suggesting that this motif might be associated with significant, distinctive functions of KMT2D. To determine whether this motif is conserved more broadly, BLASTp was performed using the “LSPPP” motif as a query to identify the human proteins in which this sequence window is found to be conserved. This analysis identifies a total of 140 human proteins that contain the “LSPPP/PPPSL” motif within their sequences, suggesting a potential significance of this conserved motif in association with any functional role in human protein regulation (Supplementary Material 3). To investigate the functional roles of these proteins, gene enrichment analysis was performed, which highlighted their involvement in key biological processes including transcriptional regulation, cell differentiation, cytoskeletal organization and epigenetic regulation. These findings raise the possibility that the “*LSPPP/PPPSL*” motif contributes to evolutionarily conserved mechanisms underlying key cellular pathways. To further assess the functional and biological significance of this motif in KMT2D, we retrieved the serine residues within the “*LSPPP*” motif of KMT2D and examined their phosphorylation status using our curated datasets.

### 3.2 Analysis of the global phosphoproteomics datasets of KMT2D

**FIGURE 2:**
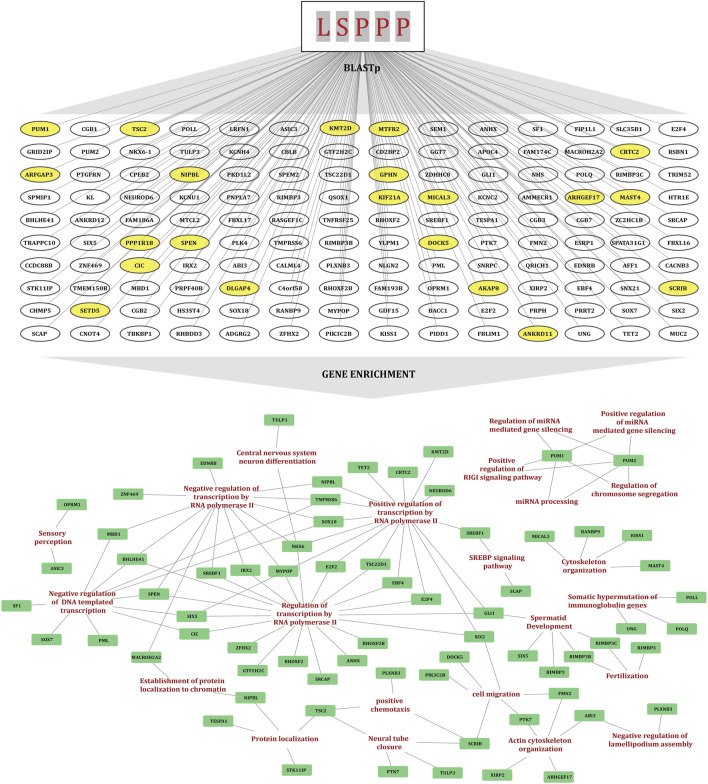
The diagram illustrating the identification of the "LSPPP" motif enriched human proteins using BLASTp and their gene set enrichment analysis. Yellow-highlighted proteins overlap with the FET high-confidence co-regulated protein dataset. A gene enrichment network is also displayed with nodes representing biological processes and proteins, connected by lines indicating interactions.

Through curation of publicly available phosphoproteomics datasets containing Class I phosphorylation sites, we identified 9,739 human qualitative profiling datasets and 1,052 quantitative differential expression datasets that reported phosphorylation events on KMT2D under diverse experimental conditions. Analysis of these datasets revealed 173 unique phosphorylation sites from qualitative profiles and 97 sites from quantitative differential datasets (Supplementary Material 1). The presence of more than 170 phosphorylation sites underscores that KMT2D is extensively regulated by kinase signaling, rendering the systematic mapping of its phospho-regulatory network through targeted *in vitro* approaches particularly challenging. To prioritize functionally relevant sites, phosphorylation events were ranked according to their frequency of detection across the curated datasets and visualized using a lollipop plot (Figure 2). This representation illustrates the distribution and relative ranking of KMT2D phosphorylation sites across both qualitative and quantitative datasets. Among them, serine 2274 (S2274), located within the conserved “*LSPPP*” motif, was identified as the most frequently phosphorylated site. Moreover, additional serine residues within this motif S468, S477, S609, S618, S663 and S672 were also consistently detected across global phosphoproteome datasets, pointing to a potentially conserved regulatory role of the “*LSPPP*” phosphomotif in KMT2D function.

#### 3.2.1 Determination of the potential co-occurrence between KMT2D phosphorylation sites

The co-occurring phosphorylation has been suggested to show potential functional associations (Li et al., 2017). To explore this in KMT2D, we systematically analyzed the co-occurrence patterns of its phosphorylation sites, with particular focus on the predominant sites to evaluate their potential regulatory significance. For this analysis, a positive co-regulation frequency greater than 3 was considered significant for positive co-occurrence, whereas a negative co-regulation frequency less than 3 was defined as significant for negative co-occurrence. Our results revealed that the most frequently detected site, S2274, showed strong positive co-regulation with several additional phosphorylation sites within KMT2D, including S2640, T2332, T3192, S3199, S3620, S3624, S4822 and S4849. These associations were consistently observed across multiple datasets. The co-occurrence plot depicting the co-regulatory patterns of KMT2D phosphorylation sites is provided in Supplementary Figure S1.

### 3.3 Protein phosphosites (PPs) that co-regulated with predominant KMT2D sites

**FIGURE 3:**
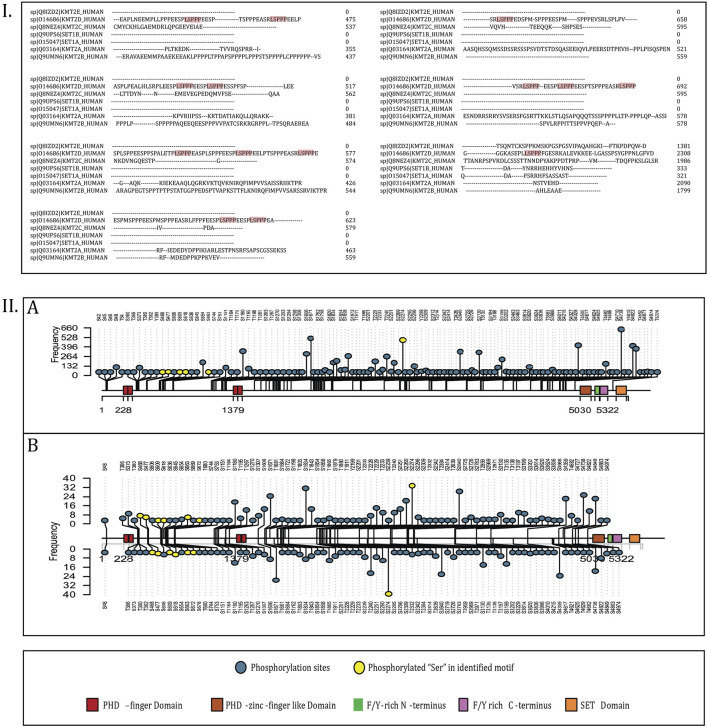
(I) Multiple sequence alignment (MSA) of KMT2 families where the “LSPPP” motif is enriched. (II) Lollipop plot visualization of the phosphorylation sites representing KMT2D. **(A)** Represents the lollipop plot of phosphorylation sites in the profiling datasets, and **(B)** represents the phosphorylation sites in differential datasets.

Co-regulated proteins often play important functional roles, and co-regulation analysis can reveal biologically relevant relationships between the proteins that may not physically interact or co-localize (Kustatscher et al., 2019). Here, we examined the PPs that tend to show consistent co-regulation with the KMT2D S2274 to identify the potential functional association between them. To ensure this, we applied strict inclusion criteria to the FET derived protein phosphosites, including (i) frequency cut off ≥10% from the total differential frequency of the predominant site for positive or negative coregulation, (ii) FET score (p-value) <0.05, (iii) consistent coregulation reported in at least three independent publications (PMID confidence) and (iv) observed across minimum of three distinct experimental conditions (code count). We identified 1,742 positively co-regulated and 88 negatively co-regulated high-confidence PPs with the KMT2D S2274. The cumulative distribution of protein phosphosites (with FET p-value <0.05) across multiple datasets that represent the positively or negatively co-regulated specific predominant phosphorylation site of KMT2D is illustrated in Figure 3. The gene enrichment analysis of the positively co-regulated proteins with KMT2D S2274 revealed its involvement in biological processes such as regulation of gene expression, chromatin remodeling, cell differentiation, and MAPK signaling pathways. The negatively co-regulated proteins are associated with processes such as negative regulation of transcription, cell division, and DNA repair (Supplementary Material 4).

### 3.4 Regulatory kinases of KMT2D phosphorylation sites

**FIGURE 4:**
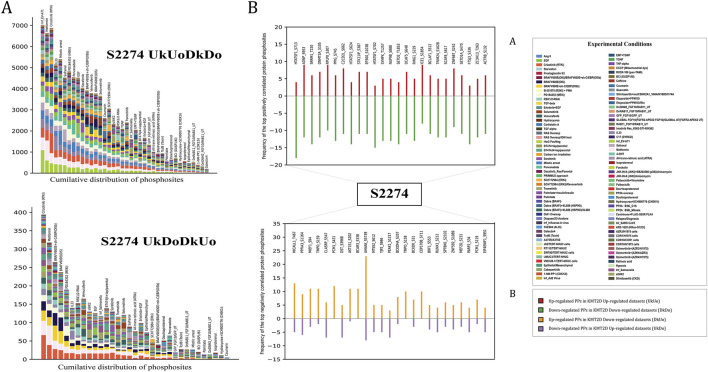
**(A)** The cumulative distribution of phosphorylation site (S2274) (with FET p-value <0.05) across multiple datasets **(B)** Top 25 high-confidence positively and negatively co-regulated PPs of KMT2D S2274.

Protein phosphorylation is one of the major post-translational events that can alter the protein function in a wide range of ways, affecting nearly all aspects of its behavior (Cohen, 2000), by means of several upstream protein kinases. Phosphorylation by these upstream protein kinases can modulate enzyme activity by either inducing or inhibiting the protein function, which can in turn influence various other biological processes (Roskoski, 2015). CDK2 was found to be the only experimentally validated *in vivo* upstream kinase of KMT2D phosphorylated at T5374 (Chi et al., 2008). MAST4 was found to be the only kinase of S2274 predicted by kinase prediction tools. Additionally, PRP4K, BUB1, MAPK14, CDK13, MAPK1, and MAPK7 were identified as the other upstream kinases (predicted by Johnson et al. (2023)) for S2274. Phosphorylation at MAPK1 (Y187 and T185), MAPK7 (S731), and MAPK14 (T180) has been associated with induced kinase activity, suggesting the activation of the corresponding MAPK signaling cascades. MAPKs (Mitogen-activated protein kinases) are crucial signaling enzymes that are specific to eukaryotes and play a role in numerous aspects of cellular regulation (Chang and Karin, 2001). The substrates of these specific kinases were also found to be positively co-regulated with S2274. p38/MAPK14 was already identified as an upstream kinase of KMT2D, where hydrogen peroxide (H2O2) induces the phosphorylation, resulting in reduced ubiquitination and degradation (Xu et al., 2020). Specifically, among the high-confidence proteins, 24 proteins are found to be the putative substrates of MAPK1, seven of MAPK14, and two of MAPK7 (Figure 4). Notably, MAPK1 (Y187 and T185), MAPK7 (S731), and MAPK14 (T180) were found to be co-regulated with S2274, suggesting a potential functional linkage between these MAPKs and the modulation of KMT2D activity. Kinases that are positively or negatively co-regulated with predominant KMT2D phosphorylation sites were identified and categorised based on the presence of phosphorylation site associated with activated kinase activity. Gene enrichment analysis further revealed that positively co-regulated proteins, including MAP2K2 (T394), IRAK1 (S131), ERBB2 (T701), RPS6KA1 (S380), RAF1 (S642, S43), EGFR (T693, S991, S1042), and EPHA2 (Y594), are associated with the MAPK signaling pathway.

#### 3.4.1 KMT2D S2274 as a potential downstream target of MEK-ERK signaling pathway

The RAS/RAF/MEK cascade triggers ERK1/2 in response to receptor tyrosine kinase (RTK) activation, which regulates cell motility, proliferation, differentiation, and survival (Chang and Karin, 2001). MAPK1 (Y187 and T185) is identified as the potential upstream kinase of KMT2D S2274 with induced kinase activity. Also, we found 24 substrates of MAPK1 to be positively co-regulated with KMT2D S2274. Interestingly, the *LSP/LTP* motif is found to be conserved for ERF (T526), ERBB2 (T701), METTL3 (S43), MYC (S77), YTHDF2 (S39), and KMT2D (S2274), which are downstream substrates of MAPK1.

**FIGURE 5:**
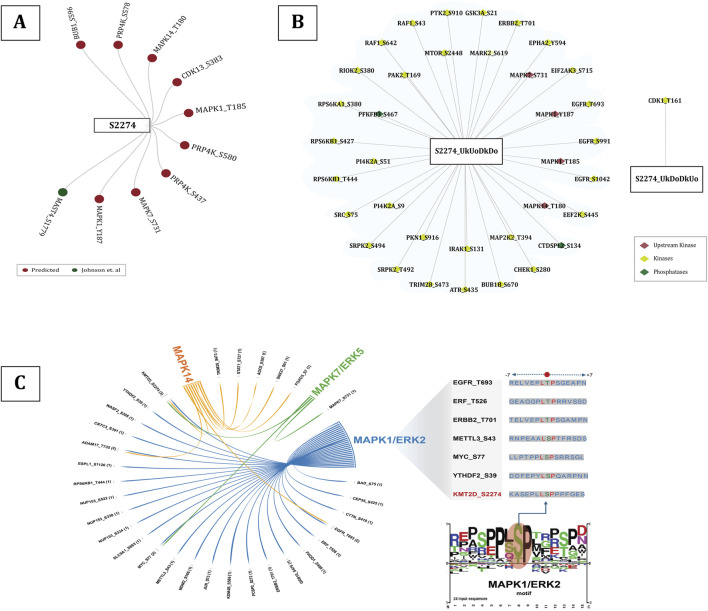
**(A)** All the upstream kinases of S2274 predicted by Johnson et al. (2023)(given in dark red color) and upstream kinases predicted by kinase prediction tools (given in dark green color), **(B)** Upregulated kinases with predominant phosphorylation sites associated with induction of their kinase activity, **(C)** Substrates of MAP kinases (MAPK1/7/14), including those specific to MAPK1/ERK2, that share the conserved *LSP/LTP* motif between KMT2D S2274.

MAP kinases phosphorylate ERF (ETS2 Repressor Factor) at T526, directly in vivo, and regulate its function (Sgouras et al., 1995). Moreover, ERF phosphorylation and export between the nucleus and the cytoplasm correlate with the levels of nuclear Erk activity (Le Gallic et al., 2004). ERK-dependent phosphorylation of ERBB2 at T701 promotes AKT dephosphorylation (Gaviraghi et al., 2020). Inhibition of this phosphorylation prolongs HER2–EGFR dimerization in a clathrin-dependent manner, resulting in increased activation of HER2 and EGFR tyrosine kinases and enhanced downstream signaling through the Akt pathway (Chen et al., 2017). ERK-mediated phosphorylation of METTL3 at S43, followed by USP5-dependent deubiquitination, stabilizes the m6A methyltransferase complex. This modification contributes to ERK-driven cancer cell activation and promotes tumorigenesis (Sun et al., 2020). ERK-dependent phosphorylation at Ser-62 promotes the recruitment of c-Myc to gamma-GCS promoters and the cellular response to oxidative stress (Benassi et al., 2006). ERK phosphorylates YTHDF2 at S39, thereby stabilizing YTHDF2 protein which is required for invasion, GBM cell proliferation, and tumorigenesis (Fang et al., 2021). Considering these studies, it is evident that ERK-mediated phosphorylation plays a pivotal role in modulating various molecular targets that are crucial for cell signaling and function. The activation of the RAF-MEK-ERK signaling cascade is initiated by the binding of various ligands to receptor tyrosine kinases (RTKs), with growth factor receptors like EGFR (Yap et al., 2011). EGFR (T693, S995, S991, S1045, S1042) is found to be upregulated with KMT2D S2274, out of which three sites (T693, S991, S1042) were identified as the activity associated sites of EGFR. Phosphorylation of EGFR at S991 (corresponding to S993 in mouse), identified through proteomic analysis, is associated with EGF stimulation and activation of downstream RAS/ERK pathway activation (Stupack et al., 2020). RAF1 is a serine/threonine kinase associated with cell growth and differentiation, functioning upstream of MEK, which phosphorylates and activates ERK (Macdonald et al., 1993). RAF1 (S43, S642), which are identified as the activity associated sites of RAF1, are positively co-regulated with KMT2D S2274. MAP2K2 (T394), the kinase that activates ERK1/2, shows positive co-regulation with the KMT2D phosphorylation site S2274. Phosphorylation of MAP2K2 at T394 enhances its activity as well as downstream ERK1/2 activation (Xu et al., 2021). The phosphorylation of KMT2D at S2274 was observed to be modulated by MAPK pathway inhibitors in melanoma cells. For instance, S2274 was found to be downregulated in response to ERK1/2 and MKK1/2 inhibitors. Specifically, it showed changes with all four inhibitors tested trametinib, selumetinib, SCH772984, and GDC0994 (Basken et al., 2018). These results strongly suggest that S2274 of KMT2D might be a downstream target of the MEK-ERK signaling cascade, which is reasonable to infer that the activation of MEK-ERK signaling may promote phosphorylation at S2274. This post-translational modification of KMT2D presumably contributes towards the dynamic regulation of the chromatin-modifying functions of KMT2D, which might link the extracellular signaling events to epigenetic modulation of gene expression. To understand the regulatory significance of S2274 in KMT2D function, we further analyzed the protein phosphosites co-regulated with S2274. Notably, our differential data revealed that S2640 and S2342 were also downregulated in response to ERK inhibitors. This further reinforces the co-occurrence between S2274 and S2640, which we had previously discussed. A detailed representation of the involvement of KMT2D S2274 in the MEK-ERK signaling pathway is represented in Figure 5.

**FIGURE 6:**
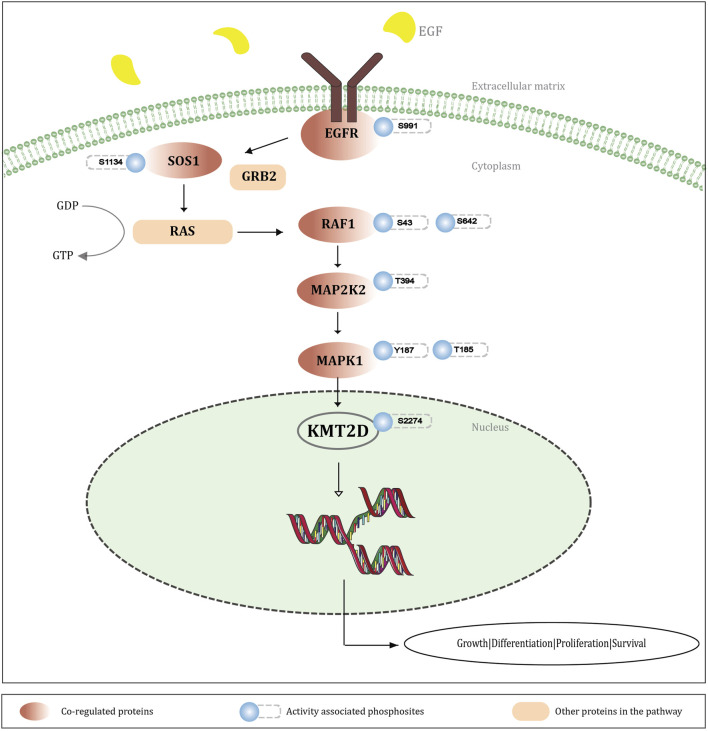
Schematic representation illustrating the integration of KMT2D S2274 phosphorylation into MEK-ERK signaling cascade. The EGF stimulation activates the EGRF-RAS-MEK-ERK pathway, leading to phosphorylation of KMT2D S2274. Co-regulated protein phosphosites and activity-associated sites within this cascade suggest that KMT2D (S2274) functions as a downstream target of MAPK signaling, affecting gene transcription and cellular processes like growth, differentiation, proliferation, and survival.

#### 3.4.2 Phosphorylation of KMT2D at S2274 in modulating cell differentiation through activation of MEK-ERK signaling

Mitogen-activated protein kinase (MAPK) cascades are important signaling pathways that regulate various cellular processes, including proliferation, differentiation, apoptosis, and stress responses, and play a critical role in the survival and development of tumour cells (Guo et al., 2020). KMT2A (paralog of KMT2D) was shown to functionally interact with one of the downstream kinases in the MAPK pathway, MSK1, enhancing H3K4 methylation via phosphorylation of histones (Wiersma et al., 2016). However, direct protein-protein interactions between KMT2D and MAPK components are still under investigation. KMT2D is expressed in adult tissues and is essential in regulating early embryonic development, metabolism, differentiation, and tumor suppression (Froimchuk et al., 2017). Our analysis identified KMT2D S2274 as a direct downstream target of MEK-ERK signaling. The RAF-MEK-ERK pathway is the most important and well-characterized signaling cascade among all the MAPK signal transduction (Guo et al., 2020; Zhang and Liu, 2002). Notably, most of the proteins associated with this pathway are upregulated with KMT2D S2274, supporting a role for KMT2D in modulating cell differentiation through activation of MEK-ERK signaling. From the gene enrichment analysis of the positively co-regulated proteins of S2274, almost 30 proteins are associated with the MAPK signaling pathway, with 23 of these mapped specifically to the KEGG MAPK signaling pathway. Positive regulation of cell differentiation was found to be the most frequently enriched Gene Ontology (GO) term in biological processes, encompassing nearly 65 of the positively co-regulated proteins with S2274 (Supplementary Figure S2).

### 3.5 Potential binary interactions of KMT2D associated with S2274 towards cell proliferation and differentiation, emphasizing its role in epigenetic regulation

Protein-protein interactions (PPIs) are essential molecular associations that reinforce a wide range of biological functions within cells. A total of 32 protein phosphosites across 10 proteins are identified to form binary interactions with S2274 of KMT2D, including six phosphorylation sites (S2239, S2260, S2640, S3199, T2332) interacting within the KMT2D itself (Supplementary Material 4). Notably, 15 phosphorylation sites of Marker of Proliferation (MKI67/KI-67), a well-established cancer biomarker associated with cell proliferation (Uxa et al., 2021), are found to be interacting with S2274. Out of the 15 phosphorylation sites, nine are localized within the KI67/Chmadrin repeat domain of MKI67, indicating that the phosphorylation of these sites might be involved in the expression and regulation of its function.

Other binary interactors that positively co-regulated with KMT2D S2274 also show association with cell differentiation. EEF1B2 (EF-1-beta and EF-1-delta), a member of the eEF1B family, influences the differentiation of BMSCs (bone marrow-derived mesenchymal stem cells) and maintains the balance between bone and fat by acting as a regulator (Feng et al., 2024). The S106 phosphorylation site of EEF1B2 present in its EF-1-beta domain is co-regulated with S2274. HERC2, an E3 ubiquitin ligase, is associated with embryonic development, particularly for neuronal and muscular functions (Elpidorou et al., 2021). KDM4B/JMJD2B, as a histone demethylase, is associated with cell growth, survival, differentiation, and gene expression. KDM4B expressions were found to be upregulated via ERK phosphorylation at S566 during glucose deprivation and this phosphorylation stabilizes KDM4B which contributes to its role in glucose uptake and cell viability, which may be regulated by epigenetically upregulating GLUT1 (Fu et al., 2018). KDM4B is also defined as an oncoprotein associated with the processes related to tumorigenesis, including cell proliferation, cell survival, and metastasis (Wang et al., 2021). KMT2D S2640 also exhibits a similar type of binary interaction as observed in S2274.

### 3.6 Implication of the S2274 phospho-mutation in the pathogenesis of kabuki syndrome

The NCBI ClinVar comprises of information about the clinical significance of genetic variants in the human genome (NCBI, 2025). It also provides genetic mutations, and the diseases associated with them at a phosphorylation site specific level. One of the predominant phosphorylation sites of KMT2D, Serine (S2274), is mutated to Leucine (L2274) or Alanine (A2274) in certain conditions, which can lead to the Kabuki syndrome (Clinvar, 2025) (Supplementary Material 5). Kabuki syndrome is a very rare congenital anomaly/intellectual disability characterized by distinctive facial features, short stature, skeletal, visceral and dermatoglyphic abnormalities, cardiac anomalies, and immunological defects (Micale et al., 2011). Mutations in KMT2D disrupt normal gene activation and lead to the features observed in Kabuki syndrome. Approximately 55%–80% of individuals with Kabuki syndrome have mutations in the KMT2D gene (Khodaeian, 2021). This mutation can disrupt the phosphorylation at the specific S2274 site, which might be associated with the poor epigenetic regulation observed in individuals affected with Kabuki syndrome. It has been previously reported that the dysfunction of known KS genes results in aberrant MEK/ERK signaling as well as disruption of F-actin polymerization and cell intercalation (Bogershausen et al., 2015). Aberrant MEK-ERK signaling and the inactivated phosphorylation of KMT2D at S2274 may contribute to the pathogenesis of Kabuki syndrome, potentially responsible for the epigenetic dysregulation and poor cell differentiation observed in affected individuals. We also analyzed the mutations across the ±7 amino acid region surrounding S2274, as these mutations may influence the phosphorylation at S2274 (Supplementary Figure S3). From these results, we can assume that there is a potential mechanistic link between disrupted phosphorylation at S2274 and the pathogenesis of Kabuki syndrome.

## 4 Limitations of the study

While this study provides insights into the phosphorylation events of KMT2D through integrated phosphoproteomics datasets, several limitations should be acknowledged. The use of publicly available mass spectrometry data presents inherent variability from differences in experimental conditions, sample types and data quality across studies. Although several inclusion and exclusion criteria were applied to filter the high-confidence co-regulated protein phosphorylation sites, the absence of experimental validation limits the confidence to confirm their direct functional significance. Although key parameters influencing detection sensitivity, such as quantification approach, mass spectrometry platform, and enrichment procedures, were accounted for in our stratification criteria, finer resolution could be achieved by systematically grouping datasets based on additional experimental factors (e.g., use of kits, SDS-PAGE fractionation). In this study, we proposed S2274 as a potential activation site of KMT2D, experimental validations will be required to functionally validate our findings. These efforts will be essential to experimentally substantiate and further elucidate the biological relevance of our findings.

## 5 Conclusion

In this study, we systematically curated and analyzed publicly available mass spectrometry–based phosphoproteomics datasets across diverse experimental conditions to generate a phosphorylation site specific landscape of KMT2D, a critical tumor suppressor involved in chromatin regulation, transcriptional control, developmental disorders, and cancer. KMT2D contributes to cell differentiation by modulating enhancer activity and controlling the expression of genes involved in differentiation processes. A key finding of our study is the identification of the “*LSPPP*” motif, a conserved sequence unique to the KMT2D among KMT family of H3K4 methyltransferases. This motif was further identified in over 140 proteins across the phosphoproteome, suggesting that it may converge upon shared regulatory mechanisms. Functional annotation of these proteins indicates that the “*LSPPP*” motif is potentially involved in processes linked to chromatin dynamics, transcriptional regulation, and cellular differentiation.

Serine 2274 (S2274), within this motif, emerged as the most frequently observed and functionally significant phosphorylation site of KMT2D. S2274 was consistently associated with co-regulated phosphorylation sites and signaling proteins, strongly suggesting that it is a downstream target of the MEK-ERK signaling cascade. Given the established role of MEK-ERK signaling in regulating cell fate and differentiation, our findings support a model in which phosphorylation at S2274 provides a mechanistic link between extracellular signaling and KMT2D-dependent epigenetic regulation. Differentially co-regulated phosphorylation sites further reinforce this association, suggesting that S2274 is a central node of KMT2D’s regulatory activity.

Importantly, the clinical relevance of this site is underscored by the observation that mutations at S2274, particularly substitutions to leucine or alanine, are associated with Kabuki syndrome, a rare developmental disorder characterized by congenital anomalies, growth impairment, immunological defects, and intellectual disability. Disruption of phosphorylation at S2274 may contribute to aberrant MEK-ERK signaling, defective chromatin regulation, and impaired cellular differentiation in affected individuals. These findings highlight the need to investigate this phosphorylation site in greater detail, suggesting further research on this aspect.

Collectively, these observations suggest that phosphorylation at S2274 plays a critical role in regulating KMT2D function in epigenetic regulation and cell differentiation through MEK-ERK mediated signaling pathway. It can be suggested that S2274 serves as an activity associated site crucial for modulating KMT2D function.

Future studies using *in vitro* kinase assays and KMT2D knockdown or knockout models will be essential to validate the regulatory role of S2274 within the conserved “*LSPPP*” motif and to define its broader significance in chromatin regulation, cell differentiation, and diseases. Our findings establish S2274 as a critical MEK-ERK dependent phosphorylation site that links extracellular signaling to epigenetic modulation highlighting its potential as both a biomarker and therapeutic target. Pharmacological modulation of this phosphorylation event may offer a precision strategy to restore or fine tune KMT2D activity in cancer and developmental syndromes.

## Data Availability

The original contributions presented in the study are included in the article/Supplementary Material, further inquiries can be directed to the corresponding authors.
