# Supplementary figures and images for: Role of histone-lysine N-methyltransferase 2D (KMT2D) in MEK-ERK signaling-mediated epigenetic regulation: a phosphoproteomics perspective
Source: Front Bioinform. 2025 Nov 18;5:1683469. doi: 10.3389/fbinf.2025.1683469 (PMC12669113; doi:10.3389/fbinf.2025.1683469)

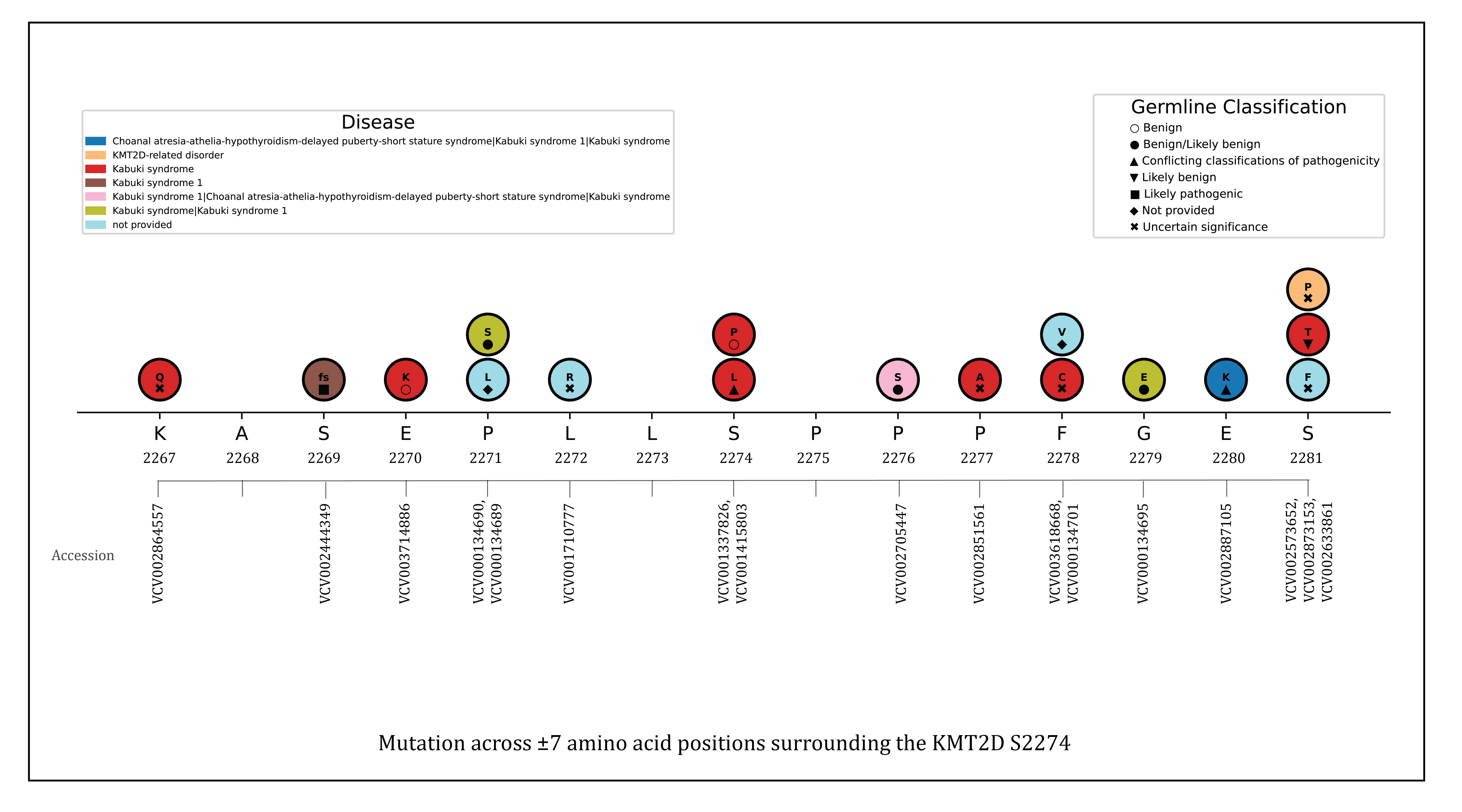

Supplement: Supplementary file 2 [file Image3.jpeg]

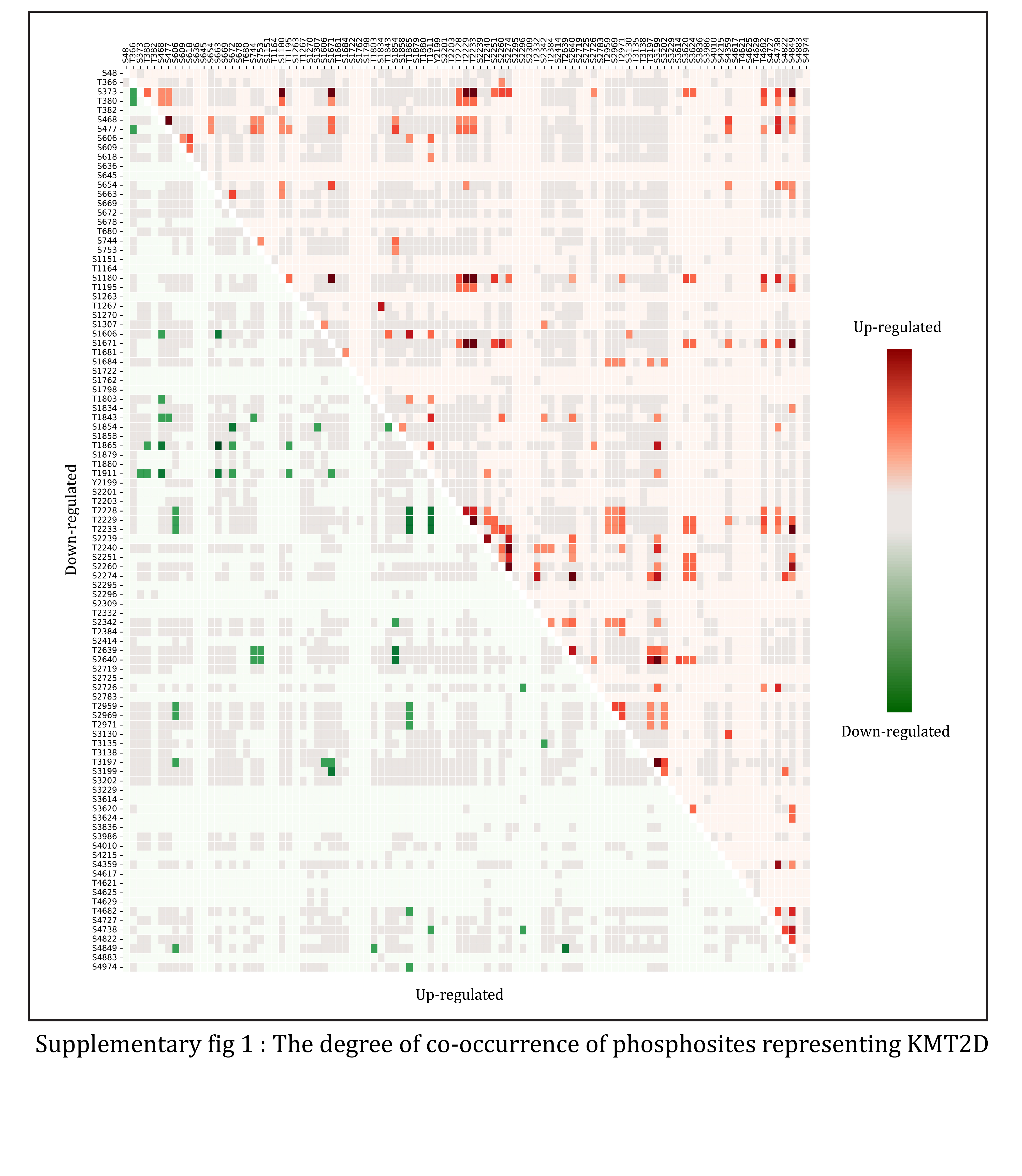

Supplement: Supplementary file 4 [file Image1.jpeg]

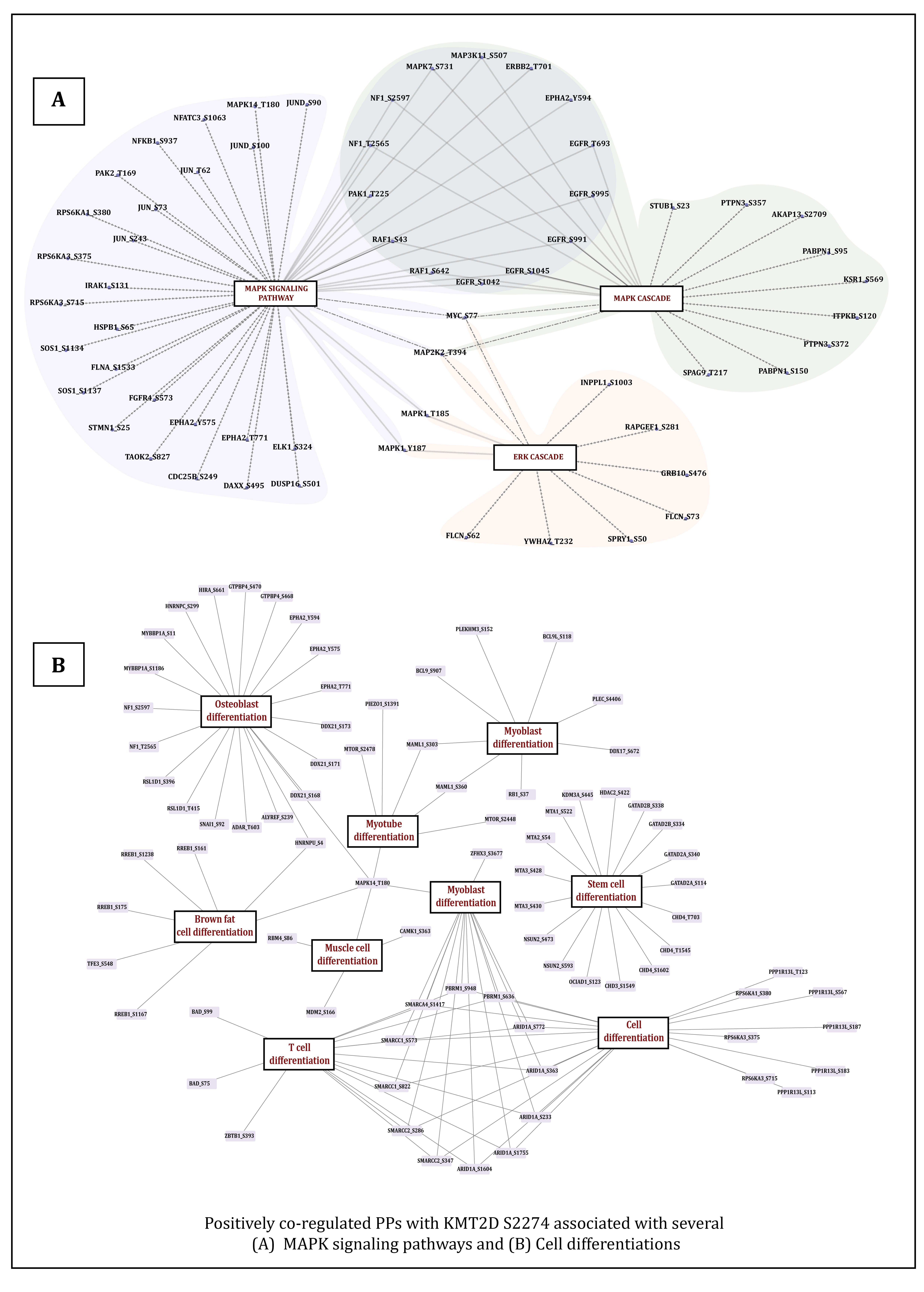

Supplement: Supplementary file 5 [file Image2.jpeg]
